# Metacognition in schizophrenia: A practical overview of psychometric metacognition assessment tools for researchers and clinicians

**DOI:** 10.3389/fpsyt.2023.1155321

**Published:** 2023-04-13

**Authors:** Vassilis Martiadis, Enrico Pessina, Fabiola Raffone, Valeria Iniziato, Azzurra Martini, Pasquale Scognamiglio

**Affiliations:** ^1^Department of Mental Health, Community Mental Health Center DS 25, ASL Napoli 1 Centro, Naples, Italy; ^2^Department of Mental Health, Community Mental Health Center, ASL Cuneo 2, Alba, Italy; ^3^Department of Mental Health, Community Mental Health Center DS 32, ASL Napoli 1 Centro, Naples, Italy; ^4^Department of Mental Health, ASL Napoli 3 Sud, San Giorgio a Cremano, Italy

**Keywords:** metacognition, assessment, schizophrenia, psychosis, metacognitive abilities, psychometry

## Abstract

Metacognition refers to the cognitive ability to control, monitor and modulate cognitive processes thus guiding and orienting behavior: a continuum of mental activities that ranges from more discrete ones, such as the awareness of the accuracy of others’ judgment, to more integrated activities, such as the knowledge of cognitive processes. Metacognition impairment in schizophrenia, which is considered a core feature of the illness, has become a growing research field focusing on a wide range of processes including reasoning, autobiographical memory, memory biases, cognitive beliefs and clinical insight. There is a well-established relationship between metacognition and schizophrenia symptoms severity, as well as between impaired metacognitive functioning and specific symptomatic sub-domains, such as positive symptoms, negative symptoms, or disorganization. The development of specific cognitive-derived psychotherapies for metacognitive deficits in schizophrenia has been ongoing in the last years. Although sharing a metacognitive feature, these treatments focus on different aspects: false or unhelpful beliefs for metacognitive therapy; cognitive biases for metacognitive training; schematic dysfunctional beliefs for cognitive behavioral therapy (CBT) for psychoses; metacognitive knowledge and sense of identity for MERIT; interpersonal ideas or events triggering delusional thinking for MIT-P. This article reviews the instruments designed to assess metacognitive domains and functions in individuals with schizophrenia, providing mental health professionals with an overview of the heterogeneous current scenario ranging from self-administered scales to semi-structured interviews, which are supported by a variety of theoretical frameworks. Future directions may address the need for more specific and refined tools, also able to follow-up psychotherapeutic-induced improvements.

## 1. Introduction

Metacognition, originally described as the ability to “think about thinking,” includes a broad range of mental processes in which the continuous integration of information leads to constructing, defining and refining a complex and evolving representation of the self and the others (1). Although formally correct, this definition may erroneously lead to consider metacognition as a singular concept. It may be more completely described as a continuum of mental activities that ranges from more discrete ones, such as the awareness of the accuracy of others’ judgment (“Introspective Accuracy”), to more integrated activities, such as the knowledge of cognitive processes, their biases and the capacity to modulate them (2). It might also be helpful to clarify that metacognition partially overlaps with the definition of other constructs such as social cognition and mentalizing, both related to the manner in which persons define ideas about themselves and the others (3). While metacognition deals with the level of integration/fragmentation of people’s sense of self and others, social cognition concerns with recognition and comprehension of others’ thoughts and emotions; mentalizing, instead, deals with the manner in which persons form their ideas in relation to their own attachment style (3).

The incremental evolution of its conceptualization led to the current concept of metacognition that is characterized both by the awareness of thoughts and by the close relationship between experiences, thoughts, and beliefs, emotions, and desires of individuals. All these elements awareness and integration contribute to constituting a dynamic, larger sense of self (3). Metacognition, however, exhibits at least four features that are not reducible to the integrated model (3). As a first point, metacognition is not by definition intentional but can include automatic proceedings. The second point is that metacognition is not confineable to one person’s mind but is intersubjective in its nature (4). The third feature is that metacognitive processes are not only linked to thoughts about oneself, but are expandable to others or larger communities (1, 5, 6). The last point is that it cannot be simply categorized as healthy/ill or intact/compromised, but it can largely vary in a multiple of both physiological and deficit degrees (3). The larger sense of self enabled by metacognition is crucial for orienting us in the inner and external world: it is a key element of human adaptive behavior as we move daily based on metacognitive insights, keeping us flexible and responsive to a complex and ever-changing reality (7). According to this model, metacognition can be more comprehensively defined as an “umbrella concept” aggregating elements ranging from discrete cognitive processes to more elaborated functions which also include neurocognitive and social cognitive abilities (8).

### 1.2. Metacognition in psychosis

Bleuler initially conceptualized that schizophrenia showed deranged goal-oriented behavior secondary to cognition disturbances making individuals unable to synthesize a stable and complex idea of self and others and of their respective place and role in reality (9). Decades of schizophrenia conceptualization have progressively moved away from this kind of cognitive alterations focusing on more discrete and easier to observe symptoms and their underlying biological, cognitive and psychological proceedings (10, 11). By using traditional neuropsychological tools, it is difficult to quantify the ability to structure the construct of self, others, and reality. This may explain a part of the deviation from Bleuler’s observations. In the last two decades, new attention on metacognition abilities stimulated the development of instruments to measure the way in which persons with severe psychiatric diseases such as psychosis are capable of combining different information into representations of the self and the others and, furthermore, contributed to bring out that metacognitive deficits constitute a core feature of these disorders. Two recent meta-analysis showed a clear relationship between metacognition, neurocognition and functional outcome (12) and a global metacognitive deficit in people suffering from schizophrenia when compared to healthy controls (13). Patients suffering from psychosis exhibit far more limited metacognitive abilities in comparison with both healthy individuals (14, 15) and people with severe medical conditions such as HIV (16): deficits are found in forming a solid sense of personal emotions and beliefs, of others’ emotions, other’s point of view and in answering to psychosocial attempts or totally eluding them. Overlapping alterations have been reported in people experiencing first episode psychosis (FEP) (17, 18). Metacognitive deficits are more evident and impactful in people with psychosis compared to bipolar disorder (19–21) depression (22, 23), anxiety disorders (24), post-traumatic stress disorder (25), substance abuse (26) and borderline personality disorder (27). Several studies investigated the neural correlates of metacognition in psychosis. One preliminary study in early stage psychosis found a significant positive correlation between metacognition abilities, prefrontal cortex and striatum gray matter density (28). A significant correlation has been found between metacognitive skills and cortical thickness in individuals with definite high psychosis risk, in specific regions such as inferior and middle frontal gyri, superior temporal cortex and the insula (29). More recent results suggest that neurophysiological bases of metacognition exhibit differential features in FEP individuals, with a critical role found in hippocampal integrity, rather than in frontal areas (30). Regarding neurofunctionality, more powerful functional connectivity in the medial prefrontal cortex, precuneus and posterior cingulus correlates with higher metacognitive performance (31); moreover, enhanced activity of rostro-lateral prefrontal cortex while performing a self-reflection task positively correlates with metacognitive performance and, in particular, with social activities (32).

Regarding psychological functions and symptomatology domains, a recent meta-analysis (33) underlined significant correlations between metacognition impairment and both symptomatic and functional outcomes. Lower metacognitive functioning was associated with more severe negative symptoms in a wide number of studies carried out in different nations and clinical settings (17, 18, 20, 21, 24, 34–36). Numerous studies showed that pronounced worsening in metacognition skills predicted more severe negative symptoms from 6 to 36 months after basal evaluation (37–40). Functional metacognition abilities, instead, predict better work functioning (41), increased physical activity (42), higher response to rehabilitation work experience (43) and more adaptive social behavior (44) regardless of general psychopathology or neuropsychological functioning, thus becoming a potential target for specific psychotherapies such as “*cognitive behavioral therapy for psychosis*”*(CBT-P)* (45), “*Metacognitive Therapy*” (46), “*Metacognitive Training*” (47), “*Metacognitive Reflection Insight Therapy*”*(MERIT)* (48) and “*Metacognitive Interpersonal Therapy for Psychosis*”*(MIT-P)* (49). Though sharing the term metacognitive, these treatments differ for epistemological basis, structure, format, hypothesized mechanisms of action and results (50).

Exploring and measuring metacognition is therefore a growing need for both researchers and clinicians involved in schizophrenia diagnosis, treatment and rehabilitation. This article aims to review evaluation instruments designed to assess metacognitive functions in individuals with schizophrenia, providing mental health professionals with a practical and useful overview of the specific available psychometric tools.

## 2. Assessing metacognition in schizophrenia

Assessing metacognitive abilities in individuals with schizophrenia and other psychosis is an essential aspect of illness comprehension and in developing research and effective treatment plans. However, this can be challenging due to the complex nature of schizophrenia, to its cognitive impairments and to the lack of insight that typically characterizes psychotic diseases. Schizophrenia can impact a person’s ability to provide accurate and consistent responses to questions. Individuals with schizophrenia may have difficulty distinguishing between reality and fantasy and/or may experience disorganized thinking, which can make it difficult for them to provide accurate and/or coherent answers. Moreover, individuals with schizophrenia may have difficulty understanding the questions and/or may have troubles in recalling information accurately, leading to inaccurate responses and impacting on the validity of the data collected. It is important to note, however, that not all individuals with schizophrenia will experience these problems to the same degree and that measures can be taken to minimize the impact of these challenges on the reliability and validity of data collection.

In the following sections, several assessment tools will be discussed, including their strengths and limitations. These instruments include both questionnaires and interviews and are summarized in Table 1.

**TABLE 1:** English language measures of metacognition.

Measure name	References	Method	Number of items/sections	Sample composition
Beck Cognitive Insight Scale (BCIS)	(52)	Self-administered scale	15 items	*n* = 150 adult inpatients diagnosed with schizophrenia, schizoaffective disorder, major depressive disorder with and without psychotic features
Indiana Psychiatric Illness Interview (IPII)	(58)	Semi-structured interview	4 sections	*n* = 33 adult outpatients diagnosed with schizophrenia or schizoaffective disorder
Metacognition Assessment Scale (MAS)	(5)	Clinician-administered scale on transcribed therapy sessions	15 items divided in 3 sections	*n* = 11
Metacognition Assessment Scale-Abbreviated (MAS-A)	(34)	Clinician-administered scale on transcribed therapy sessions	4 ordinal scales	*n* = 61 men diagnosed with schizophrenia or schizoaffective disorder
Metacognition Assessment Interview (MAI)	(61)	Semi-structured interview, derived from MAS	16 items	*n* = 175 non-clinical individuals
Metacognition Self-Assessment Scale (MSAS)	(64)	Self-administered scale, derived from MAS and MAI	18 items	*n* = 6,659 random people
Metacognitions Questionnaire (MCQ)	(72)	Self-administered scale	65 items	*n* = 25 undergraduate students *n* = 12 anxiety outpatients
Metacognitions Questionnaire-30 (MCQ-30)	(75)	Self-administered scale	30 items	*n* = 182 university students and university health service employees

### 2.1. Beck Cognitive Insight Scale (BCIS)

Recent and interesting perspectives indicate that impaired insight contributes not only to neurocognitive impairments but also to deficits in metacognition and social cognition (51), so measurement of clinical insight has become a relevant part of metacognition measurement in schizophrenia. The BCIS (52), originally developed to evaluate cognitive insight in psychosis, is today widely used in metacognition studies across the psychotic spectrum disorders, general population and other types of psychiatric disorders (52, 53). In the recent meta-analysis by Davies and Greenwood (12), 5 of 17 studies examined used the BCIS as a metacognition assessment tool, demonstrating the reliability, broad acceptance and importance of the instrument in the field. It was designed to evaluate patients’ perception of objectivity regarding their current delusional thinking, past errors, their ability to reattribute errors, and their openness to correction as well as their perception of objectiveness. A 10-item interview was initially developed and administered as a first step toward assessing cognitive insight. Questions were based on clinical observations of patients with and without psychoses as well as previous research regarding self-correction (54–56). The BCIS was so constructed reviewing these responses, within the framework of cognitive theory, adding five supplementary items and adapting the whole for self-report. The BCIS is a 15-item self-administered scale divided into a 9-item and a 6-item subscale, respectively, assessing self-reflectiveness and self-certainty. It has been shown that there is no cut-off in the BCIS scoring that distinguishes pathological from non-pathological insight in non-psychiatric individuals compared to those with psychosis. However, the researchers concluded that the BCIS can reliably discriminate between healthy individuals and people affected by psychotic disorders (57).

### 2.2. Indiana Psychiatric Illness Interview (IPII)

The IPII (58) was initially developed to assess insight in schizophrenia. On the basis that awareness of illness is inextricably tied to a person’s narrative and may therefore be incomplete or incoherent for many reasons, the authors suggested that insight assessment instruments, elaborated upon at that time, were inadequate since they did not take into account the possibility of a lack of insight as an alternative or incomplete understanding of the disease. Nowadays, since altered metacognition is considered as a disruption in the components of embodied self-experience and this disruption is more evident when individuals with schizophrenia express narratives of their lives, the IPII has become one of the principal instruments to stimulate and collect narrative production in metacognition assessment.

The IPII is a four-section semi-structured interview, taking on average from 30 to 90 min. The first section establishes a relationship with the individual who is asked to talk about the story of his/her life (“*Tell me the story of your life in as much detail as possible*”) (58). The second section deals with participant opinion about suffering or not from a mental illness (“*Do you think you have a mental illness and if so, what do you think it is?*”) (58). After an affirmative response, more specific questions are asked (“*Can you say more about your experience of mental illness in the past, about what caused these problems, how you feel about and about what is going to happen in the future?*”) (58). In the third section, the participant is asked to talk about how mental illness controls his/her life (“*To what extent and in what ways does your mental illness control your life?*”) (58). In the last part, the participant’s expectations about their future are investigated (“*What is expected to be different and what will be the same in the future?*”) (58). In the second and the fourth section, if the patient does not mention vocational, others (family, community) and/or cognitive emotional function, these are inquired about.

Interviewers should generate enough information to get a comprehensive understanding of a participant’s idea about his/her disease, rather than confirming, delimiting, or judging the participant’s narrative. Rather than focusing on content, the IPII develops narrative productions that focus on self and illness. The original version of the IPII included a scoring system for assessing narrative coherence, but the authors advise coding the resulting narratives using the metacognitive assessment scale abbreviated form (35, 59).

### 2.3. Metacognition Assessment Scale (MAS)

The authors of the MAS (5) reviewed and analyzed a large amount of clinical literature regarding disorders in the ability to recognize mental states, thus hypothesizing a modular structure in metacognitive skills, thought to be relatively independent. As a result, they subdivided the MAS into three sections: “*Understanding One’s Own Mind (UownM), Understanding Others’ Minds (UOM) and Mastery (M)*” (5), each of them constituted by their specific sub-functions (*UownM: Basic Requirements, Identification, Relating Variables, Differentiation, Integration; UOM: Basic Requirements, Identification, Relating Variables, Differentiation, Integration, Decentration; M: Basic Requirements, First-Level Strategies, Second-Level Strategies, Third-Level Strategies*) (5). “*UownM*” refers to the comprehension of one’s own mental states: *Basic Requirements* is the ability to identify one’s own mind as autonomous and different from others’ minds; *Identification* is the capacity to identify and define one’s own internal states (subdivided into A–thoughts and images, B–emotions); by means of *Relating Variables* an individual is able to explain his/her own behavior in terms of causes and/or motivations; *Differentiation* makes the individual able to recognize that mental representations are different from reality and cannot directly influence it; *Integration* is the ability to describe and discuss one’s own inner states. “*UOM*” refers to the comprehension of others’ mental states: it includes the same sub-functions described for *UownM* but relating to others’ inner states. “*M*” is the skill of analyzing and coping with representations and mental states in order to carry out effective action strategies, to complete cognitive tasks or to handle problematic mental states. “*M*” includes *First Level Strategies (MS1), Second Level strategies (MS2)*, and *Third Level Strategies (MS3)*, each one involving metacognitive tasks of growing complexity.

Therefore, the MAS assesses each individual’s metacognitive abilities as exhibited in their verbal expressions. The MAS manual contains instructions for marking single units of psychotherapy session transcriptions or of narrative transcripts obtained by means of other interviews (e.g., the IPII). The first step consists in dividing text into marked units. A text unit consists of the patient’s conversation interspersed with two interruptions from the therapist. Then the rater has to identify successes and failures in using a specific function; then successes and failures are separately assessed for each function. It is relevant to point out that the scale does not evaluate skills in terms of their presence or absence. Instead, it evaluates success or failure using that skill. In the meta-analysis by Davies and Greenwood (12) the MAS was the most used metacognition assessment tool with high reliability.

### 2.4. Metacognition Assessment Scale-Abbreviated (MAS-A)

An abbreviated version of the MAS (MAS-A) was suggested in 2005 by Lysaker et al. (34) to assess metacognition in schizophrenia patients. The MAS-A can also be used to rate therapy transcripts and most studies have used the IPII as the basis for the MAS-A ratings. The MAS metacognition focus points on functions rather than on contents (i.e., “ideas and beliefs linked to a particular mental phenomenon: beliefs about beliefs”) (34). Metacognition is described as the set of skills that allow us to understand mental phenomena and work them out in order to deal with tasks and inner states causing distress (34). In the MAS-A the initial MAS structure has been transformed into four ordinal scales. The domains represented are: “*Self-reflectivity (S)*”(34), that assesses integration or fragmentation of the sense of self through the comprehension of one’s own mental states (score range 0–9); “*Awareness of Others (O)*”(34), that measures integration or fragmentation of the sense of others through the understanding of others’ mental states (score range 0–7); “*Decentration (D)*”(34), that assesses the integrity of the sense of community through the capacity to see others and their motives in the external world (score range 0–3); “*Mastery (M)*” (34), that measures a person’s ability to deal with psychosocial challenges using one’s own mental states (score range 0–9). Items progression in “*S*,” “*O*,” and “*D*” scales follows the integration level needed for their successful engagement, with each item needing a more complex act than the antecedent one. “*S*,” “*O*,” “*D*,” and “*M*” subscales are scored in half point increments: higher scores indicate more pronounced ability to challenge with progressively more complex metacognitive acts. Subscores can be added in order to obtain a total score (range 0–28). The MAS-A has shown excellent reliability (17, 35, 42, 60) and has been applied in numerous research studies to assess metacognition impairments in different schizophrenia stages (1).

### 2.5. Metacognition Assessment Interview (MAI)

The MAI (61) is a semi-structured interview, adapted from the MAS (5, 62) which shares the same theoretical framework. The MAI assesses two main domains, “*the Self*” and “*the Other*” (61), each one made up of two dimensions: “*Monitoring*” and “*Integrating*” for “*the Self*,” “*Differentiating*,” and “*Decentration*” for “*the Other*” (61). “*Monitoring*” evaluates how an individual describes his/her actions in terms of causes and motivation. If “*Monitoring*” is deficient, the subject is unable to discriminate the reasons for his/her behavior besides recognizing or verbalizing emotions or other mental states. “*Integrating*,” the second dimension, refers to the skill to elaborate consistent descriptions of people’s mental processes and states, placing them into a meaningful order and recognizing their relevance rank. With “*Integrating*” abilities, individuals are able to understand the relation between their own mental states and behavior in different conditions. The “*Differentiating*” dimension deals with the skill to recognize the representative nature of one’s own and others’ thinking, the skill to discriminate between different categories of representations and between representations and reality. Good performance of the “*Differentiating*” function makes people flexible in formulating opinions and points of view. “*Decentration*” dimension deals with the ability to infer others’ mental states and to adopt their perspective, recognizing it as distinct from ours. “*Decentration*” means understanding others’ behaviors in relation to their own goals, beliefs and values, which might not be shareable by ourselves. A recent autobiographical, private, and interactive (in order to evaluate the other’s mental state comprehension) episode is to be described by the subject. The MAI then utilizes 4 modules, each one specific for the assessment of a single dimension. The interview takes approximately 40–50 min and is made up of 36 questions (nine for each of the four dimensions). The output of the MAI is made of 16 facets and the researcher will assign a score ranging from 1 to 5 on a Likert scale. This measure has demonstrated good inter-rater reliability and internal consistency (63).

### 2.6. Metacognition Self-Assessment Scale (MSAS)

The MSAS (64) derives from two previous metacognition scales based on the same theoretical model: the MAS (61) and the MAI (63). As a preliminary step in the development of the MSAS, the core construct dimensions were adapted from the facets developed for the MAS and the MAI, and the entire set of facets was reformulated into self-report items, thus generating a quick self-report metacognition tool (64). The MSAS, the MAS and the MAI follow the metacognitive multi-function model, a functional-focused perspective model in mind-reading skill for which metacognition is intended as a set of abilities and functions that allow discriminating mental states, reasoning about them and correctly attributing them to themselves or others (65).

The MSAS is theoretically based on mentalization and attachment theories (66, 67), theory of mind (68) metacognition (69) and metarepresentation literature (70). It is composed of three main sections: the first one deals with reflection on one’s own mental states (“*Understanding One’s Own Mind, UM*”) (64); second section deals with reflection on others’ mental states (“*Understanding Others’ Mind, UOM*” and “*Decentration, DEC*”) (64); third section deals with coping strategies to face psychological suffering and interpersonal problems (“*Mastery, M*”) (64). *UM* explores several functions as the skill to discriminate components that constitute an inner state (thoughts, images and emotions); the skill to separate classes of representations (e.g., fantasies and beliefs) and to discriminate between representations and reality; the skill to reason about different mental states and synthesize a logical description of their parts and evolution. *UOM* explores the skill to recognize emotions underlying other’s behaviors, expressions and actions, thus making possible to infer about their thinking. *DEC* captures the skill to describe others’ mental states by forming independent hypotheses and recognizing their subjectivity. *M* analyzes the use of psychological information to cope with problems of increasing levels of complexity. The MSAS includes eighteen self-rated items scored using a five-point Likert scale (ranging from 1 = never, to 5 = almost always). Higher scores suggest better ability to evaluate metacognitive abilities. Faustino et al. (71) recently described the psychometric properties of the MSAS and the relationship between metacognitive functions, meta-beliefs and cognitive fusion, concluding that the MSAS is a reliable instrument in the Portuguese population but there is still a need for a reliability and validity studies.

### 2.7. Metacognitions Questionnaire (MCQ)

The MCQ is a 65-item scale developed in 1997 (72) to measure different metacognition domains considered relevant to psychopathology according to the self-regulatory executive function model (68, 73, 74) that conceptualizes metacognitive factors involved in developing and maintaining psychological diseases. A cardinal concept is that, in psychological disorders, beliefs are made up of metacognitive elements that orient thinking and coping. This metacognitive component contributes to maladaptive emotional, thinking and behavioral styles that are partly responsible for the growth and persistence of psychological disorders.

The MCQ is made up of five related though conceptually different factors that assess the three dimensions of “*positive and negative metacognitive beliefs, metacognitive monitoring and judgments of cognitive confidence*” (72). The five metacognition domains investigated by the relative subscales are: “*Positive Beliefs (PB) about worry (Worrying helps me to avoid future problems); Negative Beliefs (NB) about the uncontrollability of thoughts and corresponding danger (My worrying is dangerous for me); beliefs concerning Cognitive Confidence (CC); negative beliefs about thoughts in general, including themes of Superstition, Punishment and Responsibility (SPR) and Cognitive Self-Consciousness (CSC) (I think a lot about my thoughts)*” (72). The possible response is rated on a four-point Likert scale whose points are defined as follows: from “do not agree” to “agree very much.” The highest scores on each subscale represent the coping strategy that the subject favors in a particular way.

### 2.8. Metacognitions Questionnaire-30 (MCQ-30)

In 2004, in order to make assessment more effective, the MCQ-65 items were revised and reduced releasing the MCQ-30 (75), a 30-element version sharing a similar factorial design, which has rapidly become the “gold standard” metacognition measurement tool. The MCQ-30 is a short multidimensional measure of metacognition beliefs, easier and cheaper to use than the 65-item version and useful to assess a few metacognitive domains considered crucial in the exploration and conceptualization of psychopathological processes. It has shown excellent psychometric properties in a large number of studies (76). The five MCQ-30 subscales (factors) reflect the original MCQ constructs: “CC (lack of confidence in memory)” (59); “PB” (understanding worrying as a useful strategy in stressful situations) (75); “CSC”; “uncontrollability and danger” (worrying understood as dangerous or uncontrollable activity) (75); “need to control thoughts” (the belief that it is important to control one’s thoughts, especially distressing thoughts) (75). The 4-point Likert response scale was the same used in the original MCQ version. Following the Metacognitions Questionnaire-Adolescent (MCQ-A) validation (76), it encouraged metacognition studies in adolescents. The MCQ-A overlaps with the MCQ-30, with slightly modified language to favor easier understanding for younger people. In addition, the development of two versions for children, the Metacognitions Questionnaire for Children (MCQ-C30) (77) and the Metacognitions Questionnaire-Child (MCQ-C) (78) boosted metacognition studies in preadolescents. These questionnaires were adapted by the MCQ-A using even more comprehensible and easier terms and language to make them suitable for younger population. Recently the Metacognitions Questionnaire-Child Revised (MCQ-CR) (79) was further simplified to make it understandable to even 7–8 years old children. While good psychometric properties emerge for the MCQ-A, only initial psychometric data support scales for younger populations in which further studies are necessary (76).

## 3. Discussion and conclusion

Metacognition deficits, today considered a core feature of schizophrenia and other psychotic disorders, need to be investigated in both clinical and research settings. Several assessment tools, reviewed in this paper, are widely used to evaluate these functions, both in a self-report and rater-administered way. Clinicians and researchers should be aware of the possibilities to accurately and reliably measure metacognition domains in order to address specific treatment options and monitor therapy improvements or illness-related progression of impairments.

It must be emphasized that metacognitive subdomains and cognitive biases, summarized in a recent systematic review and meta-analysis including 43 studies (80), such as jumping to conclusions, deviances in attributional style, theory of mind, but also overconfidence in errors and bias against disconfirmatory evidence, are thought to be implicated in the formation and maintenance of positive symptoms (81) and have become a specific target of psychotherapeutic interventions such as metacognitive training.

The present scenario of assessment instruments for metacognition abilities varies from semi-structured interviews used to obtain life narratives (IPII) that can be successively analyzed through other instruments (MAS, MAS-A), to semi-structured-interviews that can directly measure specific domains (MAI, MSAS), to progressively more specific self-administered scales (BCIS, MCQ, MCQ-30, MCQ-A, MCQ-C30, MCQ-C, MCQ-CR). Clinicians and researchers, making their choices, should take into account the specific characteristics of the instruments (theoretical basis, examined domains), the target population (age, level of insight, cognitive impairment, disease stage) and purpose of examination (basal evaluation, follow-up, case study, intervention monitoring), the time expenditure and training requirements (semi-structured-interviews vs. self-administered scales), the reliability of the single instrument, the language availability and the recognized spread and utilization of the instrument.

Future directions may address the need for more specific assessment tools, developed on the basis of the progressively refined knowledge and characterization of metacognition, neurocognition and social cognition, particularly taking into account the need to follow-up progression and improvements induced by specific metacognitive psychotherapeutic interventions.

## Author contributions

VM contributed to conception and design of the article and wrote the first draft of the manuscript. EP, FR, VI, AM, and PS wrote evaluation tools sections of the manuscript. All authors contributed to manuscript revision, read, and approved the submitted version.

## References

[1] Lysaker P Minor K Lysaker J Hasson-Ohayon I Bonfils K Hochheiser J Metacognitive function and fragmentation in schizophrenia: relationship to cognition, self-experience and developing treatments. *Schizophr Res Cogn.* (2020) 19:100142. 10.1016/j.scog.2019.100142 31828019 PMC6889776

[2] Pinkham A. Metacognition in psychosis. *J Exp Psychopathol.* (2019) 10:204380871984114. 10.1177/2043808719841146

[3] Lysaker P Cheli S Dimaggio G Buck B Bonfils K Huling K Metacognition, social cognition, and mentalizing in psychosis: are these distinct constructs when it comes to subjective experience or are we just splitting hairs? *BMC Psychiatry.* (2021) 21:329. 10.1186/s12888-021-03338-4 34215225 PMC8254212

[4] Hasson-Ohayon I Gumley A McLeod H Lysaker P. Metacognition and intersubjectivity: reconsidering their relationship following advances from the study of persons with psychosis. *Front Psychol.* (2020) 11:567. 10.3389/fpsyg.2020.00567 32269546 PMC7109331

[5] Semerari A Carcione A Dimaggio G Falcone M Nicolò G Procacci M How to evaluate metacognitive functioning in psychotherapy? The metacognition assessment scale and its applications. *Clin Psychol Psychother.* (2003) 10:238–61. 10.1002/cpp.362

[6] Dimaggio G Ottavi P Popolo R Salvatore G. *Metacognitive interpersonal therapy.* Abingdon: Routledge (2020). 10.4324/9780429350894 30458633

[7] Lysaker P Klion R. *Recovery, meaning-making, and severe mental illness: a comprehensive guide to metacognitive reflection and insight therapy.* New York, NY: Routledge (2018).

[8] Fekete Z Vass E Balajthy R Tana Ü Nagy A Oláh B Efficacy of metacognitive training on symptom severity, neurocognition and social cognition in patients with schizophrenia: a randomized controlled trial. *Scand J Psychol.* (2022) 63:321–33. 10.1111/sjop.12811 35388496 PMC9544200

[9] Bleuler E. Dementia praecox or the group of schizophrenias. *J Am Med Assoc.* (1951) 145:685. 10.1001/jama.1951.02920270079043

[10] Keshavan M Clementz B Pearlson G Sweeney J Tamminga C. Reimagining psychoses: an agnostic approach to diagnosis. *Schizophr Res.* (2013) 146:10–6. 10.1016/j.schres.2013.02.022 23498153

[11] Nemeroff C Weinberger D Rutter M MacMillan H Bryant R Wessely S DSM-5: a collection of psychiatrist views on the changes, controversies, and future directions. *BMC Med.* (2013) 11:202. 10.1186/1741-7015-11-202 24229007 PMC3846446

[12] Davies G Greenwood K. A meta-analytic review of the relationship between neurocognition, metacognition and functional outcome in schizophrenia. *J Ment Health.* (2018) 29:496–505. 10.1080/09638237.2018.1521930 30378436

[13] Rouy M Saliou P Nalborczyk L Pereira M Roux P Faivre N. Systematic review and meta-analysis of metacognitive abilities in individuals with schizophrenia spectrum disorders. *Neurosci Biobehav Rev.* (2021) 126:329–37. 10.1016/j.neubiorev.2021.03.017 33757817

[14] Hasson-Ohayon I Avidan-Msika M Mashiach-Eizenberg M Kravetz S Rozencwaig S Shalev H Metacognitive and social cognition approaches to understanding the impact of schizophrenia on social quality of life. *Schizophr Res.* (2015) 161:386–91. 10.1016/j.schres.2014.11.008 25499045

[15] Jong S Hasson-Ohayon I Donkersgoed R Aleman A Pijnenborg G. A qualitative evaluation of the effects of metacognitive reflection and insight therapy: “living more consciously”. *Psychol Psychother.* (2018) 93:223–40. 10.1111/papt.12212 30548375

[16] Lysaker P Vohs J Hamm J Kukla M Minor K de Jong S Deficits in metacognitive capacity distinguish patients with schizophrenia from those with prolonged medical adversity. *J Psychiatr Res.* (2014) 55:126–32. 10.1016/j.jpsychires.2014.04.011 24811777

[17] Trauelsen A Gumley A Jansen J Pedersen M Nielsen H Trier C Metacognition in first-episode psychosis and its association with positive and negative symptom profiles. *Psychiatry Res.* (2016) 238:14–23. 10.1016/j.psychres.2016.02.003 27086205

[18] Vohs J Lysaker P Francis M Hamm J Buck K Olesek K Metacognition, social cognition, and symptoms in patients with first episode and prolonged psychoses. *Schizophr Res.* (2014) 153:54–9. 10.1016/j.schres.2014.01.012 24503175

[19] Tas C Brown E Aydemir O Brüne M Lysaker P. Metacognition in psychosis: comparison of schizophrenia with bipolar disorder. *Psychiatry Res.* (2014) 219:464–9. 10.1016/j.psychres.2014.06.040 25017619

[20] Popolo R Smith E Lysaker P Lestingi K Cavallo F Melchiorre L Metacognitive profiles in schizophrenia and bipolar disorder: comparisons with healthy controls and correlations with negative symptoms. *Psychiatry Res.* (2017) 257:45–50. 10.1016/j.psychres.2017.07.022 28719831

[21] Lysaker P Irarrázaval L Gagen E Armijo I Ballerini M Mancini M Metacognition in schizophrenia disorders: comparisons with community controls and bipolar disorder: replication with a Spanish language Chilean sample. *Psychiatry Res.* (2018) 267:528–34. 10.1016/j.psychres.2018.06.049 29980133

[22] Ladegaard N Lysaker P Larsen E Videbech P. A comparison of capacities for social cognition and metacognition in first episode and prolonged depression. *Psychiatry Res.* (2014) 220:883–9. 10.1016/j.psychres.2014.10.005 25453639

[23] Ladegaard N Videbech P Lysaker P Larsen E. The course of social cognitive and metacognitive ability in depression: deficit are only partially normalized after full remission of first episode major depression. *Br J Clin Psychol.* (2015) 55:269–86. 10.1111/bjc.12097 26566732

[24] WeiMing W Yi D Lysaker P Kai W. The relationship among the metacognitive ability, empathy and psychotic symptoms in schizophrenic patients in a post-acute phase of illness. *Chin J Behav Med Brain Sci.* (2015) 24:128–31.

[25] Lysaker P Dimaggio G Wickett-Curtis A Kukla M Luedtke B Vohs J Deficits in metacognitive capacity are related to subjective distress and heightened levels of hyperarousal symptoms in adults with posttraumatic stress disorder. *J Trauma Dissociation.* (2015) 16:384–98. 10.1080/15299732.2015.1005331 26011671

[26] Inchausti F Ortuño-Sierra J García-Poveda N Ballesteros-Prados A. Habilidades metacognitivas en adultos con abuso de sustancias bajo tratamiento en comunidad terapéutica. *Adicciones.* (2016) 29:74. 10.20882/adicciones.719 27749965

[27] Lysaker P George S Chaudoin-Patzoldt K Pec O Bob P Leonhardt B Contrasting metacognitive, social cognitive and alexithymia profiles in adults with borderline personality disorder, schizophrenia and substance use disorder. *Psychiatry Res.* (2017) 257:393–9. 10.1016/j.psychres.2017.08.001 28826064

[28] Vohs J Leonhardt B Francis M Westfall D Howell J Bolbecker A Preliminary study of the association among metacognition and resting state EEG in schizophrenia. *J Psychophysiol.* (2016) 30:47–54. 10.1027/0269-8803/a000153

[29] Buchy L Stowkowy J MacMaster F Nyman K Addington J. Meta-cognition is associated with cortical thickness in youth at clinical high risk of psychosis. *Psychiatry Res.* (2015) 233:418–23. 10.1016/j.pscychresns.2015.07.010 26210694

[30] Alkan E Davies G Greenwood K Evans S. Brain structural correlates of metacognition in first-episode psychosis. *Schizophr Bull.* (2019) 46:552–61. 10.1093/schbul/sbz116 31776577 PMC7147593

[31] Francis M Hummer T Leonhardt B Vohs J Yung M Mehdiyoun N Association of medial prefrontal resting state functional connectivity and metacognitive capacity in early phase psychosis. *Psychiatry Res.* (2017) 262:8–14. 10.1016/j.pscychresns.2016.12.014 28208070

[32] Pinkham A Klein H Hardaway G Kemp K Harvey P. Neural correlates of social cognitive introspective accuracy in schizophrenia. *Schizophr Res.* (2018) 202:166–72. 10.1016/j.schres.2018.07.001 30077432

[33] Arnon-Ribenfeld N Hasson-Ohayon I Lavidor M Atzil-Slonim D Lysaker P. The association between metacognitive abilities and outcome measures among people with schizophrenia: a meta-analysis. *Eur Psychiatry.* (2017) 46:33–41. 10.1016/j.eurpsy.2017.08.002 28992534

[34] Lysaker P Carcione A Dimaggio G Johannesen J Nicolo G Procacci M Metacognition amidst narratives of self and illness in schizophrenia: associations with neurocognition, symptoms, insight and quality of life. *Acta Psychiatr Scand.* (2005) 112:64–71. 10.1111/j.1600-0447.2005.00514.x 15952947

[35] Nicolò G Dimaggio G Popolo R Carcione A Procacci M Hamm J Associations of metacognition with symptoms, insight, and neurocognition in clinically stable outpatients with schizophrenia. *J Nerv Ment Dis.* (2012) 200:644–7. 10.1097/nmd.0b013e31825bfb10 22759945

[36] MacBeth A Gumley A Schwannauer M Carcione A McLeod H Dimaggio G. Metacognition in first episode psychosis: item level analysis of associations with symptoms and engagement. *Clin Psychol Psychother.* (2015) 23:329–39. 10.1002/cpp.1959 25963712

[37] Hamm J Renard S Fogley R Leonhardt B Dimaggio G Buck K Metacognition and social cognition in schizophrenia: stability and relationship to concurrent and prospective symptom assessments. *J Clin Psychol.* (2012) 68:1303–12. 10.1002/jclp.21906 22886716

[38] McLeod H Gumley A MacBeth A Schwannauer M Lysaker P. Metacognitive functioning predicts positive and negative symptoms over 12 months in first episode psychosis. *J Psychiatr Res.* (2014) 54:109–15. 10.1016/j.jpsychires.2014.03.018 24725651

[39] Lysaker P Kukla M Dubreucq J Gumley A McLeod H Vohs J Metacognitive deficits predict future levels of negative symptoms in schizophrenia controlling for neurocognition, affect recognition, and self-expectation of goal attainment. *Schizophr Res.* (2015) 168:267–72. 10.1016/j.schres.2015.06.015 26164820

[40] Austin S Lysaker P Jansen J Trauelsen A Nielsen H Pedersen M Metacognitive capacity and negative symptoms in first episode psychosis: evidence of a prospective relationship over a 3-year follow-up. *J Exp Psychopathol.* (2019) 10:204380871882157. 10.1177/2043808718821572

[41] Lysaker P Dimaggio G Carcione A Procacci M Buck K Davis L Metacognition and schizophrenia: the capacity for self-reflectivity as a predictor for prospective assessments of work performance over six months. *Schizophr Res.* (2010) 122:124–30. 10.1016/j.schres.2009.04.024 19457645

[42] Snethen G McCormick B Lysaker P. Physical activity and psychiatric symptoms in adults with schizophrenia spectrum disorders. *J Nerv Ment Dis.* (2014) 202:845–52. 10.1097/nmd.0000000000000216 25380399

[43] de Jong S Renard S van Donkersgoed R van der Gaag M Wunderink L Pijnenborg G The influence of adjunctive treatment and metacognitive deficits in schizophrenia on the experience of work. *Schizophr Res.* (2014) 157:107–11. 10.1016/j.schres.2014.04.017 24908620

[44] Lysaker P Erickson M Buck B Buck K Olesek K Grant M Metacognition and social function in schizophrenia: associations over a period of five months. *Cogn Neuropsychiatry.* (2011) 16:241–55. 10.1080/13546805.2010.530470 21154067

[45] Fowler D Garety P Kuipers L Kuipers E. *Cognitive behaviour therapy for psychosis.* New York, NY: John Wiley & Sons Incorporated (1995).

[46] Fisher P Wells A. *Metacognitive therapy.* Abingdon: Routledge (2009). 10.4324/9780203881477

[47] Moritz S Andreou C Schneider B Wittekind C Menon M Balzan R Sowing the seeds of doubt: a narrative review on metacognitive training in schizophrenia. *Clin Psychol Rev.* (2014) 34:358–66. 10.1016/j.cpr.2014.04.004 24866025

[48] Hillis J Leonhardt B Vohs J Buck K Salvatore G Popolo R Metacognitive reflective and insight therapy for people in early phase of a schizophrenia spectrum disorder. *J Clin Psychol.* (2014) 71:125–35. 10.1002/jclp.22148 25557425

[49] Salvatore G Russo B Russo M Popolo R Dimaggio G. Metacognition-oriented therapy for psychosis: the case of a woman with delusional disorder and paranoid personality disorder. *J Psychother Integr.* (2012) 22:314–29. 10.1037/a0029577

[50] Lysaker P Gagen E Moritz S Schweitzer R. Metacognitive approaches to the treatment of psychosis: a comparison of four approaches. *Psychol Res Behav Manag.* (2018) 11:341–51. 10.2147/prbm.s146446 30233262 PMC6130286

[51] Lysaker P Pattison M Leonhardt B Phelps S Vohs J. Insight in schizophrenia spectrum disorders: relationship with behavior, mood and perceived quality of life, underlying causes and emerging treatments. *World Psychiatry.* (2018) 17:12–23. 10.1002/wps.20508 29352540 PMC5775127

[52] Beck A Baruch E Balter J Steer R Warman DM. A new instrument for measuring insight: the beck cognitive insight scale. *Schizophr Res.* (2004) 68:319–29. 10.1016/s0920-9964(03)00189-0 15099613

[53] Warman D Martin J. Cognitive insight and delusion proneness: an investigation using the beck cognitive insight scale. *Schizophr Res.* (2006) 84:297–304. 10.1016/j.schres.2006.02.004 16545944

[54] Gilbert D. How mental systems believe. *Am Psychol.* (1991) 46:107–19. 10.1037/0003-066x.46.2.107

[55] Lazarus R. Cognition and motivation in emotion. *Am Psychol.* (1991) 46:352–67. 10.1037/0003-066x.46.4.352 2048794

[56] Beck A Rush J Shaw B Emery G. *Cognitive therapy of depression.* New York, NY: Guilford Press (1979).

[57] Martin J Warman D Lysaker P. Cognitive insight in non-psychiatric individuals and individuals with psychosis: an examination using the beck cognitive insight scale. *Schizophr Res.* (2010) 121:39–45. 10.1016/j.schres.2010.03.028 20399611

[58] Lysaker P Clements C Plascak-Hallberg C Knipscheer S Wright D. Insight and personal narratives of illness in schizophrenia. *Psychiatry.* (2002) 65:197–206. 10.1521/psyc.65.3.197.20174 12405078

[59] Lysaker P Dimaggio G Buck K Callaway S Salvatore G Carcione A Poor insight in schizophrenia: links between different forms of metacognition with awareness of symptoms, treatment need, and consequences of illness. *Compr Psychiatry.* (2011) 52:253–60. 10.1016/j.comppsych.2010.07.007 21497218

[60] Rabin S Hasson-Ohayon I Avidan M Rozencwaig S Shalev H Kravetz S. Metacognition in schizophrenia and schizotypy: relation to symptoms of schizophrenia, traits of schizotypy and social quality of life. *Isr J Psychiatry Relat Sci.* (2014) 51:44–53. 24858634

[61] Semerari A D’Angerio S Popolo R Cucchi M Ronchi P Maffei C L’intervista per la valutazione della metacognizione (IVaM): descrizione dello strumento [The metacognition assessment interview (MAI)]. *Cognitivismo Clin.* (2008) 5:174–92.

[62] Carcione A Dimaggio G Fiore D Nicolò G Procacci M Semerari A An intensive case analysis of client metacognition in a good-outcome psychotherapy: Lisa’s case. *Psychother Res.* (2008) 18:667–76. 10.1080/10503300802220132 18815952

[63] Semerari A Cucchi M Dimaggio G Cavadini D Carcione A Battelli V The development of the metacognition assessment interview: instrument description, factor structure and reliability in a non-clinical sample. *Psychiatry Res.* (2012) 200:890–5. 10.1016/j.psychres.2012.07.015 22906953

[64] Pedone R Semerari A Riccardi I Procacci M Nicolò G Carcione A. Development of a self-report measure of metacognition: the metacognition self-assessment scale (MSAS). Instrument description and factor structure. *Clin Neuropsychiatry.* (2017) 14:185–94.

[65] Carcione A Falcone M Magnolfi F. La funzione metacognitiva in psicoterapia: scala di valutazione della metacognizione (S.Va.M.). *Psicoterapia.* (1997) 9:91–107.

[66] Allen J Fonagy P Bateman A. *Mentalizing in clinical practice.* Arlington, VA: American Psychiatric Pub (2008).

[67] Fonagy P Target M. Attachment and reflective function: their role in self-organization. *Dev Psychopathol.* (1997) 9:679–700. 10.1017/s0954579497001399 9449001

[68] Wells A. *Emotional disorders and metacognition: innovative cognitive therapy.* Chichester: Wiley (2002).

[69] Baron-Cohen S. *Mindblindness: an essay on autism and theory of mind (learning, development, and conceptual change).* Cambridge, MA: MIT Press (1995).

[70] Frith C Frith U. The neural basis of mentalizing. *Neuron.* (2006) 50:531–4. 10.1016/j.neuron.2006.05.001 16701204

[71] Faustino B Branco Vasco A Oliveira J Lopes P Fonseca I. Metacognitive self-assessment scale: psychometric properties and clinical implications. *Appl Neuropsychol Adult.* (2019) 5:596–606. 10.1080/23279095.2019.1671843 31646901

[72] Cartwright-Hatton S Wells A. Beliefs about worry and intrusions: the meta-cognitions questionnaire and its correlates. *J Anxiety Disord.* (1997) 11:279–96. 10.1016/s0887-6185(97)00011-x 9220301

[73] Wells A Matthews G. *Attention and emotion.* Hove: Psychology Press (1994). 10.4324/9781315784991

[74] Wells A Matthews G. Modelling cognition in emotional disorder: the S-REF model. *Behav Res Ther.* (1996) 34:881–8. 10.1016/s0005-7967(96)00050-2 8990539

[75] Wells A Cartwright-Hatton S. A short form of the metacognitions questionnaire: properties of the MCQ-30. *Behav Res Ther.* (2004) 42:385–96. 10.1016/s0005-7967(03)00147-5 14998733

[76] Myers S Solem S Wells A. The metacognitions questionnaire and its derivatives in children and adolescents: a systematic review of psychometric properties. *Front Psychol.* (2019) 10:1871. 10.3389/fpsyg.2019.01871 31551843 PMC6737041

[77] Gerlach A Adam S Marschke S Melfsen S. Development and validation of a child version of the metacognitions questionnaire. *Proceedings of the 38th annual congress of the European association for behavioural and cognitive therapies.* Helsinki: (2008).

[78] Bacow T Pincus D Ehrenreich J Brody L. The metacognitions questionnaire for children: development and validation in a clinical sample of children and adolescents with anxiety disorders. *J Anxiety Disord.* (2009) 23:727–36. 10.1016/j.janxdis.2009.02.013 19362445

[79] White J Hudson J. The metacognitive model of anxiety in children: towards a reliable and valid measure. *Cogn Ther Res.* (2015) 40:92–106. 10.1007/s10608-015-9725-1

[80] Penney D Sauvé G Mendelson D Thibaudeau É Moritz S Lepage M. Immediate and sustained outcomes and moderators associated with metacognitive training for psychosis. *JAMA Psychiatry.* (2022) 79:417–29. 10.1001/jamapsychiatry.2022.0277 35320347 PMC8943641

[81] Moritz S Scheunemann J Lüdtke T Westermann S Pfuhl G Balzan R Prolonged rather than hasty decision-making in schizophrenia using the box task. Must we rethink the jumping to conclusions account of paranoia? *Schizophr Res.* (2020) 222:202–8. 10.1016/j.schres.2020.05.056 32507550

